# Membrane fluidity homeostasis is required for tobramycin-enhanced biofilm in *Pseudomonas aeruginosa*

**DOI:** 10.1128/spectrum.02303-23

**Published:** 2024-02-27

**Authors:** Audrey David, Ali Tahrioui, Rachel Duchesne, Anne-Sophie Tareau, Olivier Maillot, Magalie Barreau, Marc G. J. Feuilloley, Olivier Lesouhaitier, Pierre Cornelis, Emeline Bouffartigues, Sylvie Chevalier

**Affiliations:** 1 Unité de recherche Communication Bactérienne et Stratégies Anti-infectieuses, CBSA UR4312, Université de Rouen Normandie, Normandie Université, Evreux, France; 2 Fédération de Recherche Normande Sécurité Sanitaire, bien être, Aliment Durable (SéSAD), Evreux, France; Hong Kong University of Science and Technology, Hong Kong, Hong Kong

**Keywords:** tobramycin, biofilm, SigX, membrane fluidity

## Abstract

*Pseudomonas aeruginosa* is an opportunistic pathogen, which causes chronic infections, especially in cystic fibrosis (CF) patients where it colonizes the lungs via the build-up of biofilms. Tobramycin, an aminoglycoside, is often used to treat *P. aeruginosa* infections in CF patients. Tobramycin at sub-minimal inhibitory concentrations enhances both biofilm biomass and thickness *in vitro*; however, the mechanism(s) involved are still unknown. Herein, we show that tobramycin increases the expression and activity of SigX, an extracytoplasmic sigma factor known to be involved in the biosynthesis of membrane lipids and membrane fluidity homeostasis. The biofilm enhancement by tobramycin is not observed in a *sigX* mutant, and the *sigX* mutant displays increased membrane stiffness. Remarkably, the addition of polysorbate 80 increases membrane fluidity of *sigX*-mutant cells in biofilm, restoring the tobramycin-enhanced biofilm formation. Our results suggest the involvement of membrane fluidity homeostasis in biofilm development upon tobramycin exposure.

**IMPORTANCE:**

Previous studies have shown that sub-lethal concentrations of tobramycin led to an increase biofilm formation in the case of infections with the opportunistic pathogen *Pseudomonas aeruginosa*. We show that the mechanism involved in this phenotype relies on the cell envelope stress response, triggered by the extracytoplasmic sigma factor SigX. This phenotype was abolished in a *sigX*-mutant strain. Remarkably, we show that increasing the membrane fluidity of the mutant strain is sufficient to restore the effect of tobramycin. Altogether, our data suggest the involvement of membrane fluidity homeostasis in biofilm development upon tobramycin exposure.

## INTRODUCTION

*Pseudomonas aeruginosa* is an opportunistic pathogen causing infections in immuno-compromised persons and the main etiological agent of chronic infections worsening the state of the health of cystic fibrosis (CF) patients (1). *P. aeruginosa* infections are difficult to treat and develop from an initial colonization into intermittent and, subsequently, chronic infections. This is mainly due to the survival of the pathogen in biofilm-like microcolonies, which develop during chronic infections (2–4). Bacterial biofilms are defined as highly structured ecosystems, in which microorganisms are attached to the neighboring cells on the surfaces, and embedded into a complex matrix composed of exopolysaccharides, extracellular DNA (eDNA), proteins, and outer membrane (OM) vesicles (5, 6). Their development is a multistep process that requires a significant change in bacterial physiology and metabolism and results in increased tolerance to exogenous stress, including antibiotics (5, 7, 8). Tolerance toward antibiotics, i.e., the ability to survive under treatment without developing resistance, has gained importance these last years, notably because it was shown to facilitate resistance evolution (9). Bacterial tolerance within biofilms can originate from the low concentration of antibiotics or the intermittent antibiotic exposure experienced by the cells. For example, the biofilm exopolysaccharide layers may slow the antibiotic diffusion (10). Bacteria in the inner layers of biofilms may also experience lower concentrations and a more gradual increase in antibiotic levels than those in outer layers (10, 11), during which time an adaptative response may emerge (12, 13). Exposure to antibiotics at levels below the minimal inhibitory concentrations (sub-MIC) was shown to induce biofilm development in common clinical pathogens such as *Staphylococcus aureus*, *Enterococcus faecalis, Escherichia coli*, and *P. aeruginosa* (14–19). This phenomenon occurs in response to antibiotics of various classes, including aminoglycosides (19–23), quinolones (24), and tetracyclines (21, 23), suggesting that the molecular mechanism leading to biofilm enhancement upon antibiotic exposure might be at least partly non-specific.

The aminoglycoside tobramycin is a frontline drug currently used in the treatment of *P. aeruginosa* in CF and other diseases (25) and was shown to induce biofilm development in *P. aeruginosa* exposed to sub-MIC concentrations (19). Recently, we elucidated the adaptive mechanisms shaping the tobramycin-enhanced biofilm formation (19). Our observations support a potential adaptive mechanism, in which the 4-hydroxy-2-alkylquinolines [2-heptyl-4-quinolone (HHQ) and 2-heptyl-3-hydroxy-4(1H)-quinolone (PQS)], representing one of the three quorum sensing pathways of *P. aeruginosa* (26), and the PrrF small RNAs involved in iron homeostasis regulation (27) were identified as key players in the changes in the biofilm architecture (19). While this molecular mechanism led to eDNA release presumably resulting from cell death (28) and consequently to the increased matrix and biofilm production, a remaining question however relies on the direct effect of tobramycin on the bacterial cell. Tobramycin crosses the bacterial OM via the process of self-promoted uptake (29, 30). It competitively binds to negatively charged lipopolysaccharides, displacing divalent cations, and disrupting the integrity of the OM, thus causing increased OM permeability (31). This complex process also involves cytoplasmic membrane traversal driven by membrane potential and ribosome disruption, leading to the production of membrane-damaging mistranslated polypeptides (16).

The bacterial envelope is the first barrier that is in direct contact with the antibiotic. It must be monitored to be repaired and modified in response to environmental assaults. The cell envelope stress response gathers the pathways that can sense envelope damages or defects and encompass all regulatory events to mitigate stress, enabling the bacterial cell to maintain its envelope integrity (32–34). In *P. aeruginosa*, the cell wall stress response is mainly triggered by the two extracytoplasmic function sigma (ECFσ) factors AlgU and SigX (32), both of which are involved in biofilm formation (33, 34). AlgU is activated in response to heat shock, low shear stress, or peptidoglycan alterations originating from D-cycloserine treatment (35–38). Noticeably, expression of *sigX* was also shown to be increased in these two latter conditions (35, 39). The ECFσ SigX is involved in membrane homeostasis, via *de novo* fatty acid biosynthesis, leading to increased membrane fluidity (40–42). It responds to high concentrations of sucrose (40), in the absence of the major porin OprF (41), cold shock (37), and several membrane-acting compounds (42–45). Altogether, the ECFσ SigX was thus suggested to respond to membrane tensions and to regulate membrane fluidity (32, 37, 46, 47). In view of these data, we hypothesized that the interactions between tobramycin and the OM may lead to a cell envelope stress response that might be involved in biofilm development.

## MATERIALS AND METHODS

### Bacterial strains and growth conditions

The strains used in this study are listed in Table S1. Bacteria were grown aerobically on a rotary shaker (180 rpm) at 37°C in Luria-Bertani (LB) broth containing 171 mM (10 g.L^−1^) NaCl, and their growth was followed by OD_580nm_ determination. Biofilms were grown in LB for 24 h in the static condition in microtiter plates at 37°C. When required, *P. aeruginosa* strains were grown in LB containing 0.8 µg.mL^−1^ of tobramycin (LBT, Sigma Aldrich, Saint-Louis, MI, USA) or various concentrations of polysorbate 80 (PS80) (LBPS80, Sigma Aldrich).

### General DNA procedures

Restriction enzymes, T4 DNA ligase, and alkaline phosphatase were purchased from New England Biolabs (Ipswich, MA, USA) and used according to the manufacturer’s instructions. PCR assays were carried out with 1 µg of *P. aeruginosa* strain H103 chromosomal DNA, 20 pmol of each primer (Table S1), and Taq DNA polymerase (Roche Molecular Biochemicals). When necessary, PCR products and plasmids were purified with the QIAquick or QIAprep Spin Miniprep kits (QIAGEN), respectively. *E. coli* and *P. aeruginosa* were transformed by electroporation (Gene Transformer GTF100, Savant) as previously described (48, 49).

### RNA extraction and quantitative RT-PCR

Total RNAs from three independent cultures were isolated by the hot acid-phenol method as previously described (40), followed by rigorous treatment with Turbo DNA-*free* kit (Invitrogen) according to the manufacturer. Synthesis of cDNAs and RT-qPCR were achieved as previously described (50), using the primers listed in Table S1. The expression level of the mRNAs was calculated by comparing the threshold cycle (Ct) of target genes with the control sample group and the relative quantification data were determined with the 2^−ΔΔCt^ method (51) using DataAssist software (Applied Biosystems).

### Construction of a *gfp* reporter strain of *sigX* promoter region activity

The non-stable green fluorescent protein (GFP) (asv)-encoding gene and the ribosome-binding site were amplified by PCR on pjAB113 vector (52) using taataaGAATTCAGAGGAGAAATTAAGCATGCGT and taataaAAGCTTAACCGAGCGTTCTGAACAAA primers, generating a 859 bp fragment. The *Eco*RI-*Hin*dIII-digested PCR product was inserted into pBBR1-MCS5 (53), yielding pBBGFP. The insert was verified by DNA sequencing (Beckman Coulter Genomics). To monitor *sigX* transcription, the 686 bp upstream of the *sigX* transcriptional start site (40) was amplified by PCR using primers taataaGGATCCGAGTCGCTCGGCCTGCA and taataaGAATTCGTGGAACAGCTCCGAGTGCG. The resulting 686 bp fragment and pBBGFP were digested by *Eco*RI and *Bam*HI, then ligated together yielding pBB686GFP. The insert was verified by DNA sequencing (Beckman Coulter Genomics). After transformation of *P. aeruginosa* H103 either by pBBGFP empty vector or by pBB686 reporting the activity of *sigX* promoter region, the strains were grown in biofilm for 24 h in LB or LBT medium before being observed by Confocal Laser Scanning Microscopy (CLSM) as indicated elsewhere.

### Biofilm formation assays and CLSM

Overnight planktonic cultures were diluted to an OD_580nm_ value of 1 in physiological water solution (0.9% NaCl) and spotted on glass-bottom microplates. After 2 h at 37°C without shaking, planktonic bacteria were removed and LB medium with or without 0.8 µg.mL^−1^ of tobramycin was added. Biofilm cultures were grown for 24 h at 37°C in static conditions. Prior to image acquisition, biofilm cells were rinsed with physiological water solution, labeled with fluorescent dyes, and observed by CLSM. Biofilm cells were stained by adding 5 µM of SYTO 9 green fluorescent nucleic acid stain (Invitrogen, Thermo Fisher Scientific; excitation at 488 nm and emission from 500 to 550 nm). To assay *sigX* promoter region activity, bacteria were labeled using the SYTO 62 Red Fluorescent Nucleic Acid Stain (Thermo Fisher), and the green fluorescence was related to the activity of the *sigX* promoter region. The live/dead fluorescent staining was performed using the LIVE/DEAD BacLight Bacterial Viability Kit (Thermo Fisher). Cells were labeled with a mixture (vol/vol) of component A (SYTO 9, 1.67 mM/propidium iodide, 1.67 mM) and component B (SYTO 9, 1.67 mM/propidium iodide 18.3 mM) according to the recommendations of the supplier. The CLSM observations were carried out with a Zeiss LSM710 (Carl Zeiss Microscopy, Oberkochen, Germany) using a 63× oil immersion objective. Images were taken every micrometer throughout the whole biofilm depth. For visualization and processing of three-dimensional (3D) images, the Zen 2.1 SP1 zen software (https://www.zeiss.com/microscopy/int/downloads/zen.html) (Carl Zeiss Microscopy, Oberkochen, Germany) was used. The average and maximum thicknesses (μm) and biovolumes (μm^3^.μm^−2^) of biofilms were measured using the COMSTAT software (54) (http://www.imageanalysis.dk/). At least three image stacks from each of the three independent experiments (nine stacks in total) were used for each analysis.

### Biofilm assays

*P. aeruginosa* H103 biofilms were grown on 96-well polystyrene microplates (Thermo Fisher Scientific, Nunc, Waltham, MA, USA). Strains were inoculated at OD_580nm_ = 1 with or without 0.8 µg.mL^−1^ of tobramycin or different concentrations of PS80 in LB medium. The biofilm cultures were incubated for 24 h at 37°C without shaking. After incubation, cell growth was determined by OD_580nm_. Biofilm amount was measured by discarding the medium, rinsing the wells with physiologic water solution, and staining any bound cells with crystal violet at 0.1% for 15 min. The dye was dissolved in 30% (vol/vol) of acetic acid, and A_595nm_ was recorded using the Spark 20 M multimode microplate reader controlled by Spark Control software Version 2.1 (Tecan Group Ltd., Crailsheim, Germany). Three independent biological assays were performed, and OD_595nm_ values were normalized with cell mass measured at OD_580nm_.

### Fluorescence anisotropy assays

*P. aeruginosa* H103 was grown in LB liquid medium or as a biofilm on 24-well polystyrene microplates (Thermo Fisher Scientific, Nunc). Strains were inoculated at OD_580nm_ = 1 with or without 0.8 µg.mL^−1^ of tobramycin (Sigma Aldrich) or 0.1% of PS80 (Sigma Aldrich) in LB medium. After incubation for 24 h at 37°C with (planktonic cultures) or without shaking (biofilm), cells were harvested by resuspension in sterile MgSO_4_ buffer (10 mM), washed twice, and suspended in the same buffer to OD_580nm_ = 0.1. Membrane fluidity was measured using the probe 1,6-diphenyl-1,3,5-hexatriene (4 µM) (DPH, Sigma Aldrich) solubilized in tetrahydrofuran (Sigma Aldrich). Tetrahydrofuran alone was used as a control for each sample. Measurement was performed as previously described (45).

### Statistical analysis

Statistical significance was evaluated using the Prism GraphPad online tool (https://www.graphpad.com/quickcalcs/ttest1/). The data were statistically analyzed using a two-sample unpaired *t*-test to calculate *P* values. The mean with SEM was calculated and plotted.

## RESULTS

### Tobramycin increases *sigX* expression and activity and membrane fluidity in *P. aeruginosa* planktonic cells

**Fig 1:**
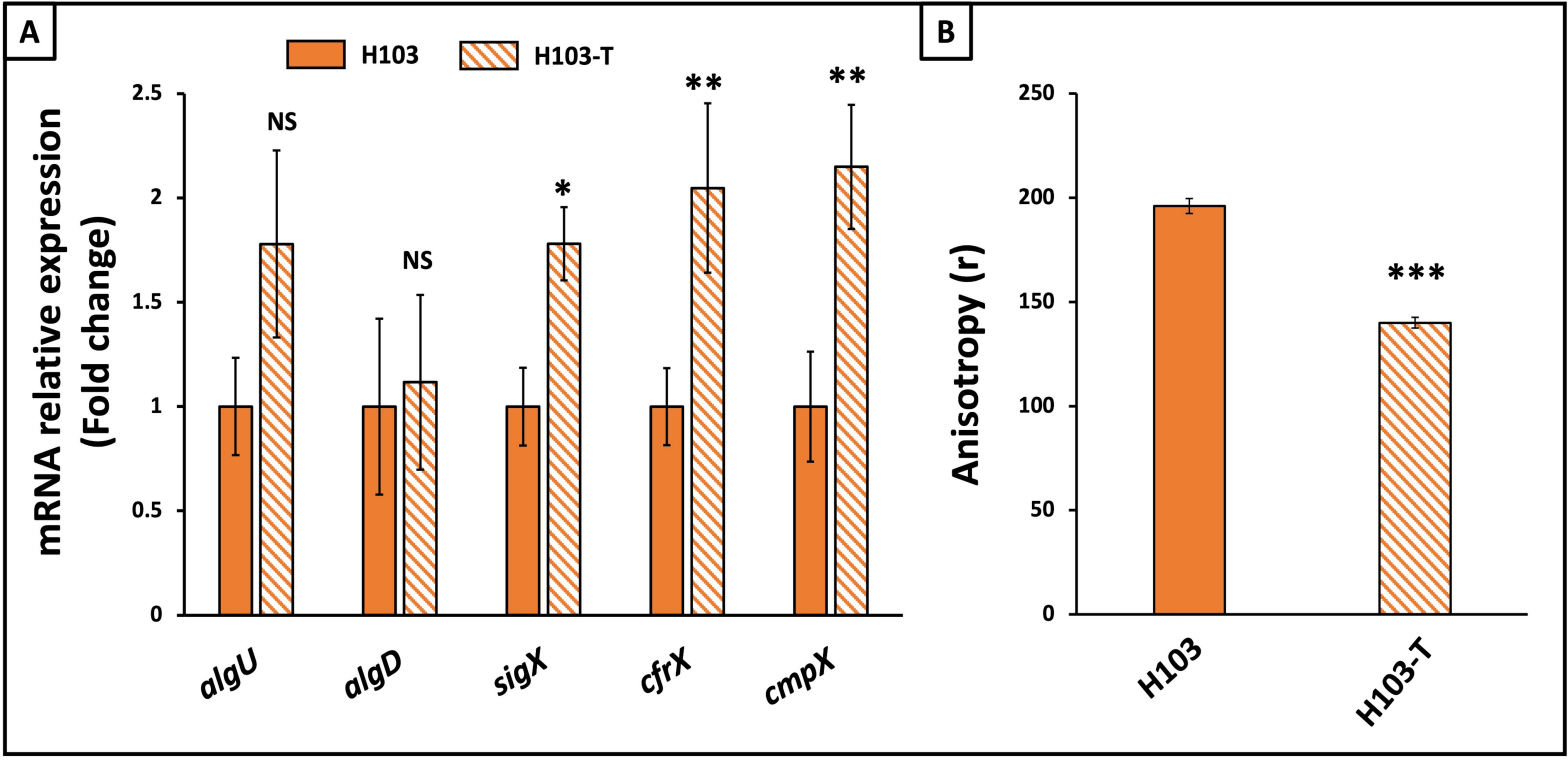
Tobramycin increased *sigX* expression and activity and membrane fluidity in *P. aeruginosa* planktonic cells. (**A**) Relative mRNA expression of *sigX, algU,* and their specific targets *cfrX*, *cmpX*, and *algD*, respectively, in *P. aeruginosa* exposed to 0.8 µg.mL^−1^ of tobramycin (H103-T) compared to the relative expression of mRNA levels in the control condition (H103), after 24 h of growth (180 rpm). (**B**) Fluorescence anisotropy (**R**) was measured after insertion of the DPH probe on planktonic cells of H103 without (orange) or with (orange hatches) 0.8 µg.mL^−1^ of tobramycin. Quantifications have been obtained from at least three independent experiments, and error bars represent the standard error of the means. Statistics were achieved by student’s *t*-test between H103 and H103-T: ***P = 0.0001 to 0.001, **P = 0.001 to 0.01; *P = 0.01 to 0.05; NS, not significant, *P* ≥ 0.05.

In a previous study, we showed that *P. aeruginosa* H103 increased biofilm formation upon exposure to tobramycin at concentrations ranging from 0.5 to 1 µg.mL^−1^, with the highest effect observed at 0.8 µg.mL^−1^ (19). To evaluate the effect of tobramycin on *P. aeruginosa* cell envelope stress response, we assessed the expression and activity of the two major ECFσ cell envelope stress response AlgU and SigX. Thus, transcript-level expression of *sigX* and *algU* and of some of their specific targets (37, 41) was evaluated using RT-qPCR from RNAs extracted from 24 h planktonic cell culture of *P. aeruginosa* exposed to 0.8 µg.mL^−1^ of tobramycin. No significant expression difference was observed for *algU* and its target gene *algD*, upon tobramycin exposure, suggesting that the AlgU-related pathway is not mainly affected in this condition (Fig. 1A). By contrast, expression of *sigX*, as well as of two of its target genes *cfrX* and *cmpX* (37), was significantly increased by about twofold, suggesting that SigX was activated upon exposure to tobramycin (Fig. 1A). Since SigX was involved in increasing membrane fluidity, notably through its role in *de novo* fatty acid biosynthesis (46), we assayed the effect of tobramycin exposure on *P. aeruginosa* membrane fluidity. This assay was performed by fluorescence anisotropy using the DPH fluorescent probe (55), which diffuses into the hydrophobic regions of the lipid bilayer. A decreased fluorescence anisotropy of the membranes is associated with a decreased order of the phospholipid hydrocarbon chains, thus reflecting increased membrane fluidity (55). Accordingly, fluorescence anisotropy assays showed an enhancement of membrane fluidity when cells were exposed to 0.8 µg.mL^−1^ of tobramycin (Fig. 1B). Altogether, these data suggest an involvement of SigX and/or membrane fluidity in response to exposure to a sub-MIC of tobramycin.

### Tobramycin exposure increases *sigX* expression and membrane fluidity in *P. aeruginosa* biofilm cells

**Fig 2:**
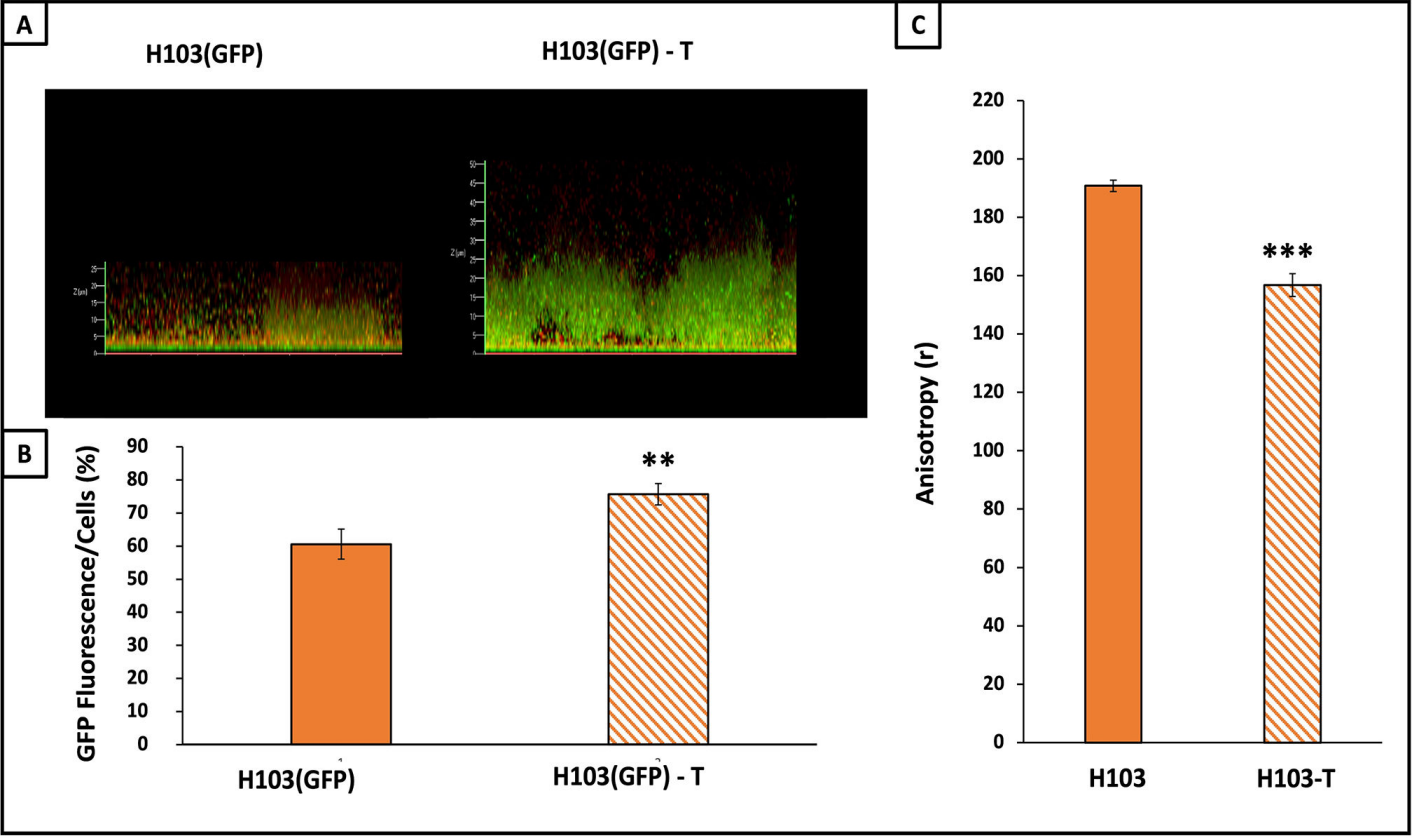
Tobramycin increased *sigX* expression and membrane fluidity in *P. aeruginosa* biofilm cells. (**A**) CLSM 3D images of 24-h-old biofilms after biomass labeling by SYTO 62 (red) of *P. aeruginosa* H103 with or without 0.8 µg.mL^–1^ of tobramycin. *sigX* promoter activity is followed by GFP fluorescence (green). Images show representative data from at least three independent biofilm assays. (**B**) The ratio between GFP fluorescence and cell biomass as evaluated by COMSTAT 2 analyses. The error bars represent the standard error of the means (SEMs) and are the result of the analysis of three views of each of the three independent biological assays. (**C**) Fluorescence anisotropy (R) was measured after the insertion of the DPH probe on biofilm cells of H103 without (orange) or with (orange hatches) 0.8 µg.mL^−1^ of tobramycin. Quantifications have been obtained from at least three independent experiments and error bars represent the standard error of the means. Statistics were achieved by student’s *t*-test between strains with or without tobramycin: ***P = 0.0001 to 0.001; **P = 0.001 to 0.01.

Because biofilm is a multicellular structure, in which cells display a huge heterogeneity in terms of physiology and metabolism, studying gene expression of sessile cells is consequently highly variable (56, 57). Thus, to study *sigX* expression within the biofilm, we constructed a transcriptional fusion between the promoter region of *sigX* and the GFP-encoding gene and the control strain, lacking the *sigX* promoter region, which was transferred into *P. aeruginosa* H103 (see the “Experimental procedures” section). Biofilm cultures of *P. aeruginosa* H103 harboring *sigX::gfp* construction were grown in LB medium without or with tobramycin at sub-MIC (0.8 µg.mL^−1^). After 24 h, the biofilms formed by these strains were labeled with the SYTO 62 Red Fluorescent probe, observed under CLSM, and analyzed by COMSTAT2 (Fig. 2A). Both green fluorescence reporting SigX activity and red fluorescence corresponding to the bacterial biomass were evaluated within the biofilms (Fig. 2B). Using this strategy, we showed increased activity of *sigX* promoter region within the biofilm cells upon tobramycin exposure (Fig. 2A and B), suggesting that *sigX* was more expressed in response to tobramycin within the biofilms. As a control, no significant fluorescence was detected when *P. aeruginosa* was transformed with the empty vector during biofilm growth in the absence or presence of tobramycin (data not shown), showing that the increased fluorescence observed was related to enhanced *sigX* expression. Since membrane fluidity was affected upon tobramycin exposure in planktonic cells, the same assay was conducted in biofilm growth conditions. As shown in Fig. 2C, tobramycin exposure of *P. aeruginosa* H103 reduced moderately but significantly reduced the DPH-associated fluorescence anisotropy, suggesting that this antibiotic increases *P. aeruginosa* H103 membrane fluidity also in biofilm growth condition, according to the increased *sigX* promoter activity in response to tobramycin.

### Tobramycin exposure did not lead to increased biofilm or membrane fluidity in a *sigX*-mutant strain

**Fig 3:**
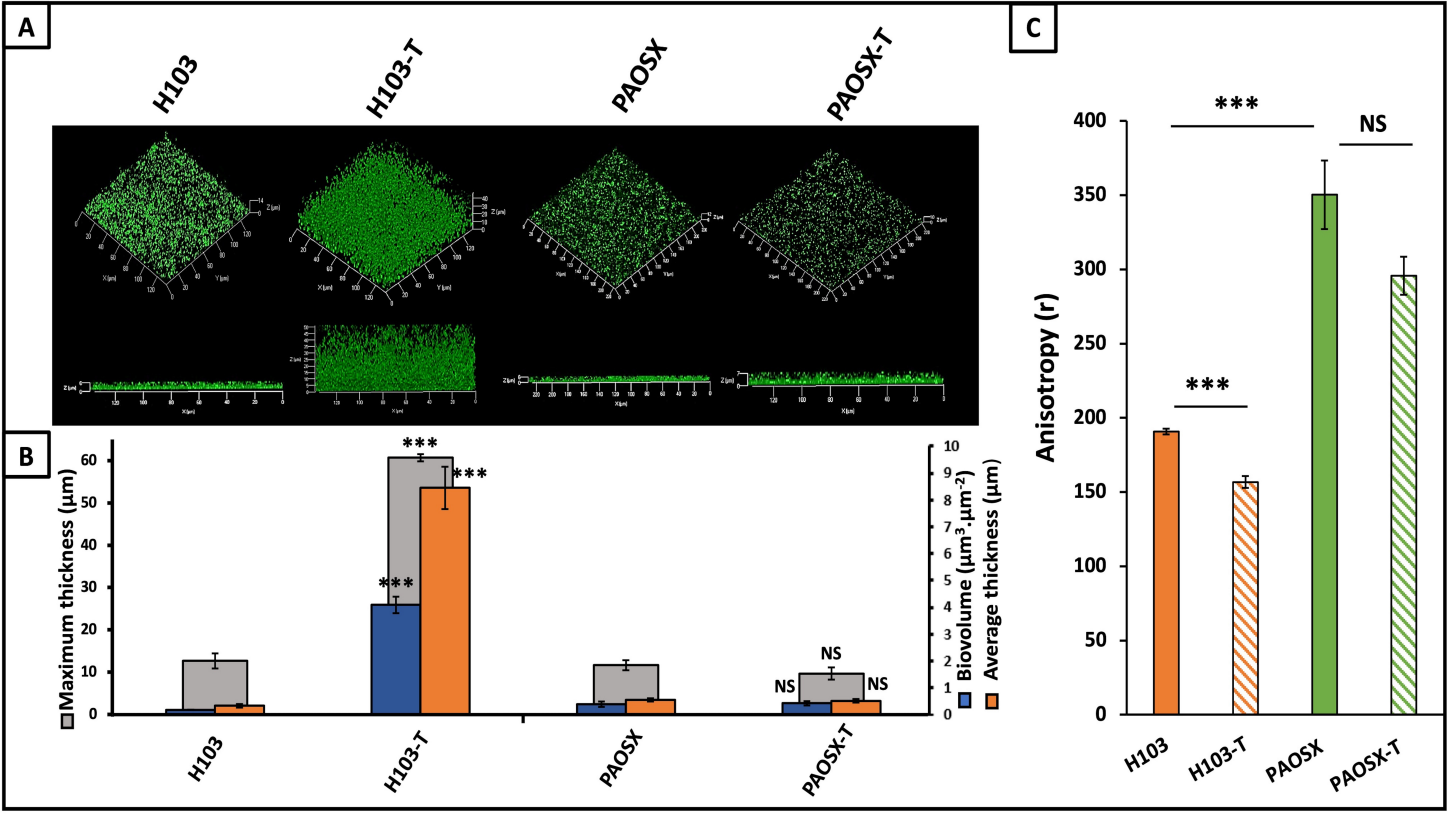
SigX and membrane fluidity may be involved in biofilm enhancement in response to tobramycin. (**A**) CLSM 3D images of 24-h-old biofilms after biomass labeling by SYTO 9 green in *P. aeruginosa* H103 and isogenic *sigX*-deletion-mutant PAOSX with or without 0.8 µg.mL^−1^ of tobramycin. Images show representative data from at least three independent biofilm assays. (**B**) COMSTAT 2 analyses were performed to determine maximum thicknesses (μm), average thicknesses (μm), and biovolumes (μm^3^.μm^−2^). The error bars represent the standard error of the means (SEMs) and are the result of the analysis of three views of each of the three independent biological assays. (**C**) Fluorescence anisotropy (R) was measured after the insertion of the DPH probe on biofilm cells of H103 (orange) PAOSX *sigX*-mutant strain without (green) or with (orange and green hatches) 0.8 µg.mL^−1^ of tobramycin. Quantifications have been obtained from at least three independent experiments and error bars represent the standard error of the means. Statistics were achieved by student’s *t*-test between strains with or without tobramycin. ***P = 0.0001 to 0.001; NS, not significant, *P* ≥ 0.05.

To get further insights into the involvement of the ECFσ SigX, biofilms of *P. aeruginosa* H103 and its isogenic *sigX*-deletion mutant PAOSX (40) were observed by CLSM and analyzed by COMSTAT2 in terms of biovolumes and average and maximal thicknesses, as previously described (54). Beforehand, E-tests were carried out to verify PAOSX susceptibility to tobramycin. As shown in Fig. S1, the PAOSX-mutant strain was slightly more susceptible to tobramycin than *P. aeruginosa* H103 since no growth was observed for concentrations over 1 µg.mL^−1^ for PAOSX and 1.5 µg.mL^−1^ for H103. Upon tobramycin treatment, *P. aeruginosa* H103 displayed increased biofilm formation (Fig. 3A). The biovolumes, average thickness, and maximum thickness, were 40-, 26-, and 5-fold higher than when biofilms were grown without tobramycin (Fig. 3B), according to our previous study (19). By contrast, PAOSX displayed a biofilm that seems similar to H103 (Fig. 3A), a phenotype that was confirmed by COMSTAT2 analyses since no significant difference was detected in terms of biovolume and average and maximum thicknesses between the two strains (Fig. 3B). Interestingly, tobramycin exposure did not enhance biofilm formation of PAOSX-mutant strain, a result confirmed by COMSTAT2 analyses of CLSM images since no differences could be evidenced, neither at the thickness nor at the biovolume levels (Fig. 3B). We then questioned whether the absence of biofilm increase in PAOSX could be due to cell death within the biofilm upon tobramycin exposure. Live-dead labeling assays were performed on biofilms developed by *P. aeruginosa* H103 and PAOSX in the absence or presence of tobramycin, and CLSM images were analyzed using COMSTAT2. The proportion of live and dead cells was similar between H103 and PAOSX strains in LB medium and after exposure to tobramycin (Fig. S2), showing that the increased biofilm impairment observed in the *sigX* deletion mutant is not due to increased cell death.

The effect of tobramycin on PAOSX membrane fluidity was then assayed. In a previous study, the PAOSX-mutant strain was shown to display a more rigid membrane than *P. aeruginosa* H103 when grown in planktonic conditions (47). We show herein that this phenotype was similar when bacteria were grown in a biofilm lifestyle (Fig. 3C), given that an increased fluorescence anisotropy of the membrane-embedded DPH is reflecting increased membrane stiffness (55). Furthermore, exposure to tobramycin did not affect significantly PAOSX-mutant membrane fluidity (Fig. 3C). Altogether, these data suggest an involvement of SigX and/or membrane fluidity in response to exposure to a sub-MIC concentration of tobramycin.

### Tobramycin-enhanced biofilm is associated with changes in membrane fluidity

**Fig 4:**
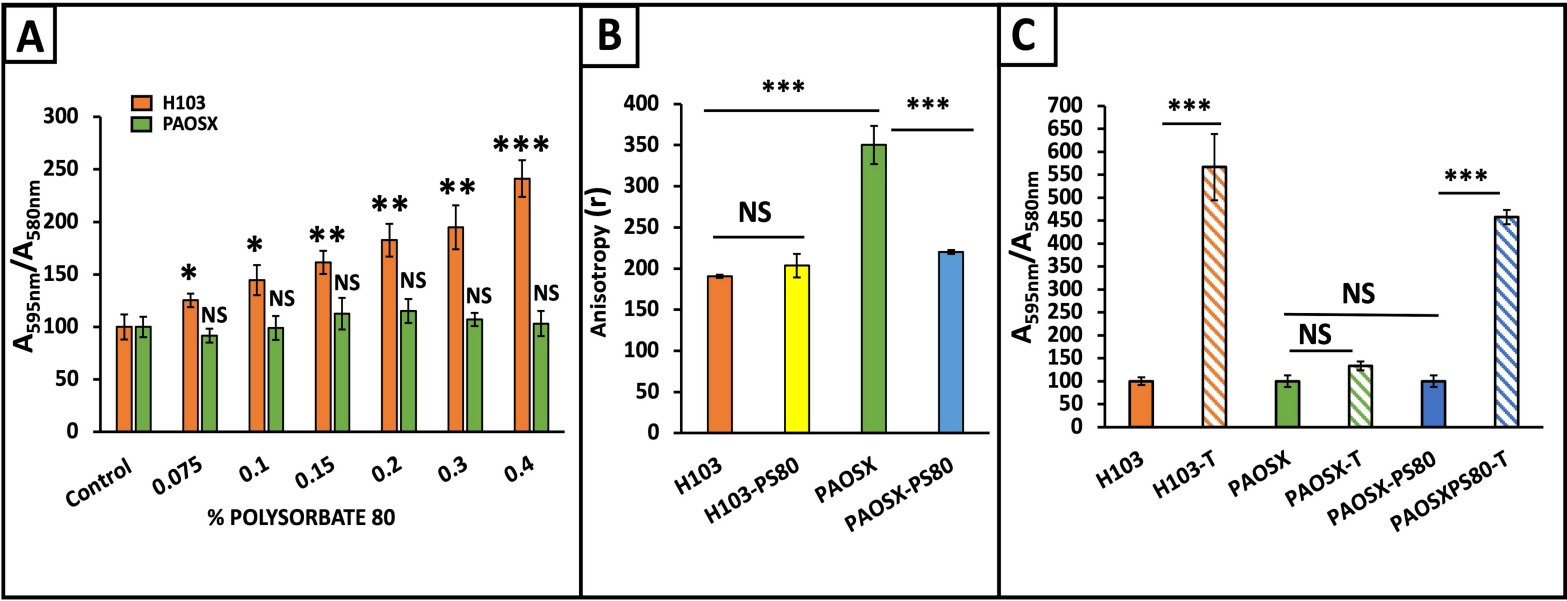
Increased membrane fluidity restores biofilm growth in PAOSX in response to tobramycin. (**A) and (C**) Quantification of biofilm after crystal violet (CV) staining by absorbance measurement at 595 nm. OD_595nm_ values were normalized to the biomass measured at OD_580nm_. Three independent biological assays were performed, and the error bars represent the standard error of the means (SEMs). Statistics were achieved by student’s *t*-test between strains with and without 0.8 µg.mL^−1^ of tobramycin or PS80. ***P = 0.0001 to 0.001; **P = 0.001 to 0.01; *P = 0.01 to 0.05; NS, not significant, *P* ≥ 0.05. (b) Fluorescence anisotropy (R) was measured after the insertion of the DPH probe into H103 and PAOSX grown in biofilm with (orange and yellow) or without (green and blue) 0.1% of PS80. Four independent biological assays were performed. The error bars represent the standard error of the means. Statistics were achieved by student’s *t*-test between strains with or without tobramycin. ***P = 0.0001 to 0.001, **P = 0.001 to 0.01; *P = 0.01 to 0.05; NS, not significant, *P* ≥ 0.05.

Since SigX is involved in short-chain fatty acid biosynthesis, and thus in membrane fluidity, we next assessed whether the tobramycin-dependent biofilm enhancement could be related to membrane fluidity. To this aim, the membrane-acting agent PS80 was used to alter *P. aeruginosa* membrane fluidity as previously observed (37, 47), and its effect on biofilm formation was assayed. As shown in Fig. 4A, increasing concentrations of PS80 caused an increased biofilm formation of *P. aeruginosa* H103, but not of PAOSX strain, mimicking thus the effects of tobramycin. We therefore selected the concentration of 0.1% of PS80 to further assay the membrane fluidity of strains by fluorescence anisotropy. In a previous study, we demonstrated that PS80 could restore the membrane fluidity of the PAOSX-mutant strain to the wild-type level when the bacteria were grown in a planktonic lifestyle (47). When *P. aeruginosa* H103 cells were assessed in a sessile lifestyle (biofilm formation), PS80 had no effect on its membrane fluidity but significantly increased that of the PAOSX-mutant strain to a level close to that of the wild-type strain (Fig. 4B). In addition, adding tobramycin at 0.8 µg.mL^−1^ to H103 and PAOSX previously treated with PS80 did not alter their membrane fluidity since no significant differences could be measured by fluorescence anisotropy assays between these conditions (Fig. S3). Altogether, these data suggest that membrane fluidity could be linked to increased *P. aeruginosa* biofilm formation.

To support this hypothesis, we then assayed the biofilm formation in *P. aeruginosa* H103 and PAOSX strains in microtiter plates (Fig. 4C). The obtained results showed that tobramycin supplementation leads to increased biofilm formation of the wild-type strain (H103) by 5.7-fold, while PAOSX biofilm was unchanged (Fig. 4C). The treatment of PAOSX with PS80 increased its membrane fluidity; however, biofilm formation in this condition was not affected. Remarkably, tobramycin exposure of PAOSX previously treated with PS80 resulted in a 4.6-fold increase in biofilm formation (Fig. 4C). Altogether, these data show that the biofilm increase upon tobramycin treatment could be restored in a *sigX*-mutant strain by increasing its membrane fluidity prior to sub-MIC antibiotic treatment, suggesting that membrane fluidity rather than SigX by itself may be involved in this phenotype.

## DISCUSSION

In this study, we found that sub-MIC of tobramycin increased *P. aeruginosa* biofilm formation by a mechanism that seems to be related to membrane fluidity. Exposure to sub-MIC of tobramycin was shown previously to increase biofilm formation of *P. aeruginosa*, a phenotype that has been already observed for various bacterial species in response to several antibiotic classes (15, 16, 19–23, 58). Such features could represent an advantage to the cells to resist the antimicrobial activity of tobramycin since the effect of this antibiotic was shown to be confined to the upper parts of *P. aeruginosa* biofilm (10), leading to reducing its concentration within the biofilm. The glucosyltransferase NdvB that allows the production of cyclic-β- (1,3)-glucans was shown to be important for *P. aeruginosa* PA14 resistance to tobramycin when grown in biofilm. These periplasmic glucans were proposed to sequester tobramycin, thus interfering with the passage of antibiotics from the periplasmic space to their site of action in the cytoplasm (59). The mechanism appeared to be biofilm-specific due to the specific expression of the *ndvB* gene in biofilm cells compared to planktonic cells (59, 60). Tolerance mediated by *ndvB* was shown to be due to drug sequestration by these cyclic periplasmic glucans, as well as due to a role in the activation of ethanol oxidation genes (59, 60). Recently, the adaptive mechanisms shaping the tobramycin-enhanced biofilm formation were partly elucidated (19), with a major involvement of the alkylquinoline pathway of the QS and the PrrF small RNAs in the changes in the biofilm architecture (19). Matrix modifications including increased eDNA abundance levels and PrrF small regulatory RNAs were also observed (19). A remaining question, however, relies on the effect of tobramycin on the bacterial cell to increase biofilm formation.

Since tobramycin and other aminoglycosides have both bactericidal effects through membrane disruption and impaired protein synthesis (29–31, 61), we hypothesized that the interaction between bacteria and the antibiotic could generate a cell envelope stress response. The latter is mainly triggered by the ECFσ factors AlgU and SigX, which both are involved in biofilm development (32, 38). Based on RT-qPCR experiments in planktonic growth conditions, we show herein that the AlgU-related pathway was not mainly affected, whereas expression and activity of SigX were enhanced upon tobramycin exposure. Previous studies have identified several conditions, in which *sigX* but not *algU* was expressed. This was the case noticeably when *P. aeruginosa* was subjected to membrane interactive compounds or physicochemical conditions, leading to a membrane stiffening, such as a cold shock (37) or hypo-osmolarity (40, 62, 63). Using a strategy based on a transcriptional fusion between the *sigX* promoter region and the GFP-encoding gene, we show that tobramycin exposure increased the activity of the promoter, suggesting that *sigX* was more expressed within the biofilm in response to this antibiotic. Accordingly, a transcriptomic approach, performed to identify genes differentially expressed during biofilm growth (64), shows that *cfrX*, one of SigX targets (37), was increased in response to tobramycin (10 µg.mL^−1^), suggesting that SigX was more expressed in this condition. Interestingly, PrrF1, whose expression was shown to be increased in response to tobramycin (19), was suggested to belong to SigX regulon, as shown by RNA sequencing (65).

The ECFσ SigX is a master regulator leading to the production of short-chain fatty acids (46), allowing membrane fluidity homeostasis (37, 38, 45–47). Indeed, while the lack of SigX results in membrane stiffness (47), its overproduction leads to a more fluid membrane (46). In the present study, in addition to biofilm development, we show that sub-MIC tobramycin leads to an increase in *P. aeruginosa* membrane fluidity. Noticeably, tobramycin did affect neither biofilm formation nor membrane fluidity in the *sigX*-mutant strain. Despite a tendency of tobramycin to increase the membrane fluidity of *sigX* mutant, this effect was however not significant since the membrane of the mutant remains stiff in the presence of tobramycin as compared to that of the wild-type strain. Taken together, our data suggest a relationship between membrane fluidity and the tobramycin-related biofilm increases in *P. aeruginosa*.

To explore this hypothesis, the non-ionic detergent PS80 was used to fluidify the membrane of *P. aeruginosa* cells independently of SigX activity (66). PS80 has previously been shown to increase the membrane fluidity in planktonic *sigX*-mutant cells (47), and likewise, it decreases their membrane stiffness when grown in biofilm to a level that was similar to that of wild-type cells. While increasing the *sigX*-mutant membrane fluidity, however, PS80 exposure did not increase biofilm formation, suggesting that this detergent is not sufficient to enhance biofilm formation in the absence of SigX. However, PS80 promotes *P. aeruginosa* H103 biofilm formation, according to a previous study (67), without modifying the membrane fluidity. Remarkably, exposure of the *sigX*-mutant cells treated with PS80 to sub-MIC of tobramycin led to restoring the enhancement of biofilm formation, suggesting that SigX by itself is not directly required to this phenomenon, but rather indirectly, by increasing the membrane fluidity. Thus, we propose that tobramycin may exert its effect on relatively fluid membranes that could lead to increased biofilm formation. In the same way, this would explain why PS80 may promote biofilm formation in *P. aeruginosa* wild-type strain but not in the *sigX* mutant. It has been reported that the membranes of established biofilm cells are more rigid than planktonic cells (68, 69). Moreover, switching from planktonic to biofilm lifestyles has been recently correlated to decreased membrane fluidity (69). However, our study suggests that enhancement of biofilm formation by sub-MIC of tobramycin requires membrane fluidity homeostasis. It is thus possible that the membrane stiffness of the *sigX*-mutant cells prevents further biofilm expansion triggered by tobramycin.

Although antibiotics are administered in amounts sufficient to inhibit bacterial growth *in vitro*, antibiotic effectiveness can be reduced within the infected airways of CF patients noticeably because of the biofilm growth of *P. aeruginosa* that limits antibiotic delivery to bacteria (70, 71). It is therefore likely that bacteria are exposed to sub-minimal antibiotic concentrations. Overall, the present study paves the way for other studies to be conducted on a possible relationship between membrane fluidity homeostasis and biofilm formation. In addition, exposure to sub-MIC of other antibiotics, such as quinolones (24) and tetracycline (21, 23), enhances *P. aeruginosa* biofilm formation. Conversely, some other antibiotics, such as polymyxin B, carbenicillin, and chloramphenicol, do not impact biofilm development (20). The effect of the combination of antibiotics, such as tobramycin and beta-lactam, both at a low dose, on biofilm formation would be of interest. Further insights into the role of membrane fluidity/stiffness on biofilm formation should be investigated since control of membrane fluidity may be also important for full-scale biofilm development.

## SUPPLEMENTAL MATERIAL

Fig. S1 spectrum.02303-23-s0001.tiff E-test assays on H103 and PAOSX.

Fig. S2 spectrum.02303-23-s0002.tiff Presence of tobramycin does not affect cell viability in PAOSX.

Fig. S3 spectrum.02303-23-s0003.tiff Tobramycin exposure has no effect on membrane fluidity of H103 and PAOSX previously treated with PS80.

Table S1 spectrum.02303-23-s0004.docx List of strains and primers used in this study.

ASM does not own the copyrights to Supplemental Material that may be linked to, or accessed through, an article. The authors have granted ASM a non-exclusive, world-wide license to publish the Supplemental Material files. Please contact the corresponding author directly for reuse.
