# Supplementary figures and images for: Membrane fluidity homeostasis is required for tobramycin-enhanced biofilm in Pseudomonas aeruginosa
Source: Microbiol Spectr. 2024 Feb 27;12(4):e02303-23. doi: 10.1128/spectrum.02303-23 (PMC10986583; doi:10.1128/spectrum.02303-23)

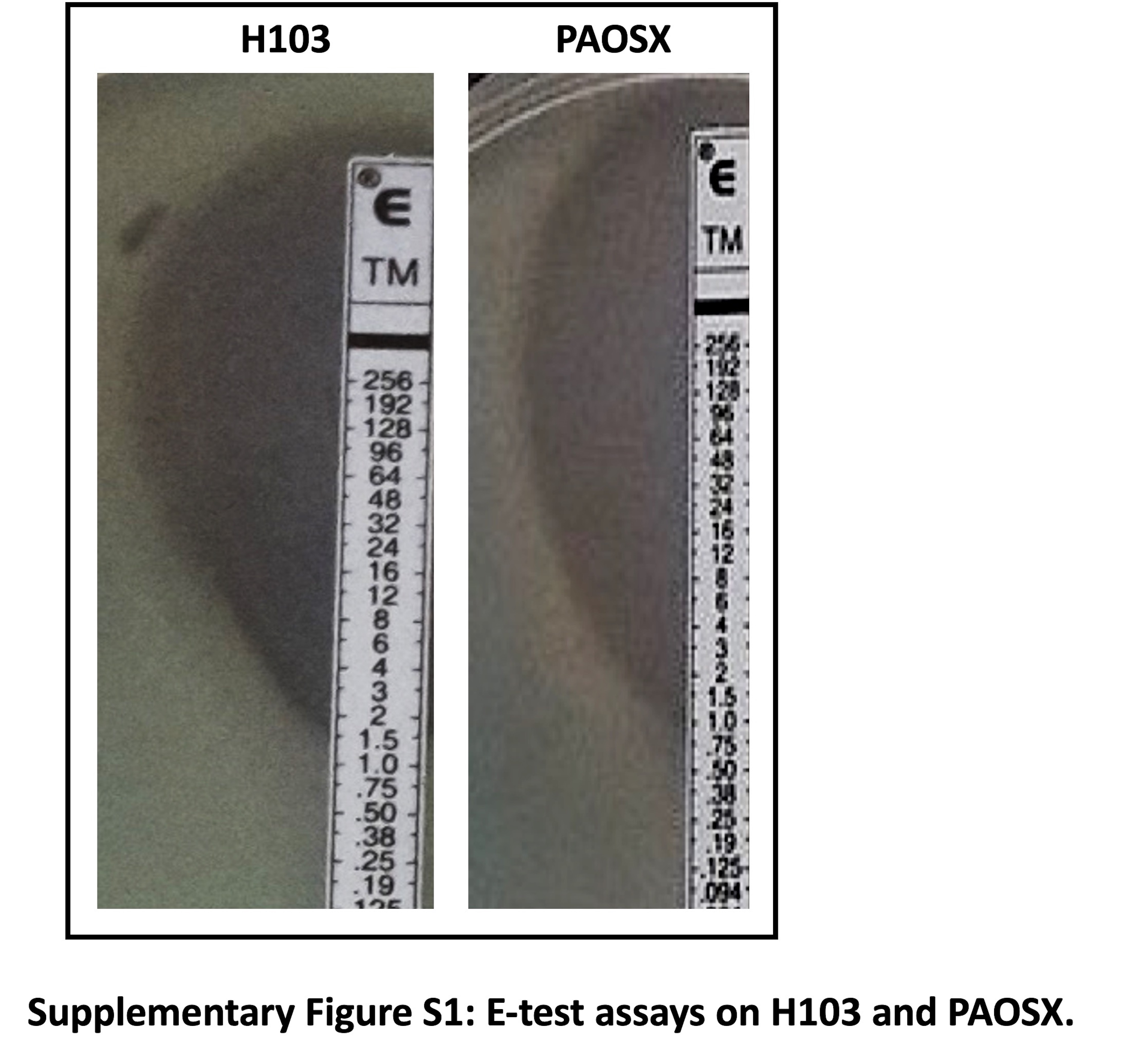

Supplement: Fig. S1 — E-test assays on H103 and PAOSX. [file spectrum.02303-23-s0001.tiff]

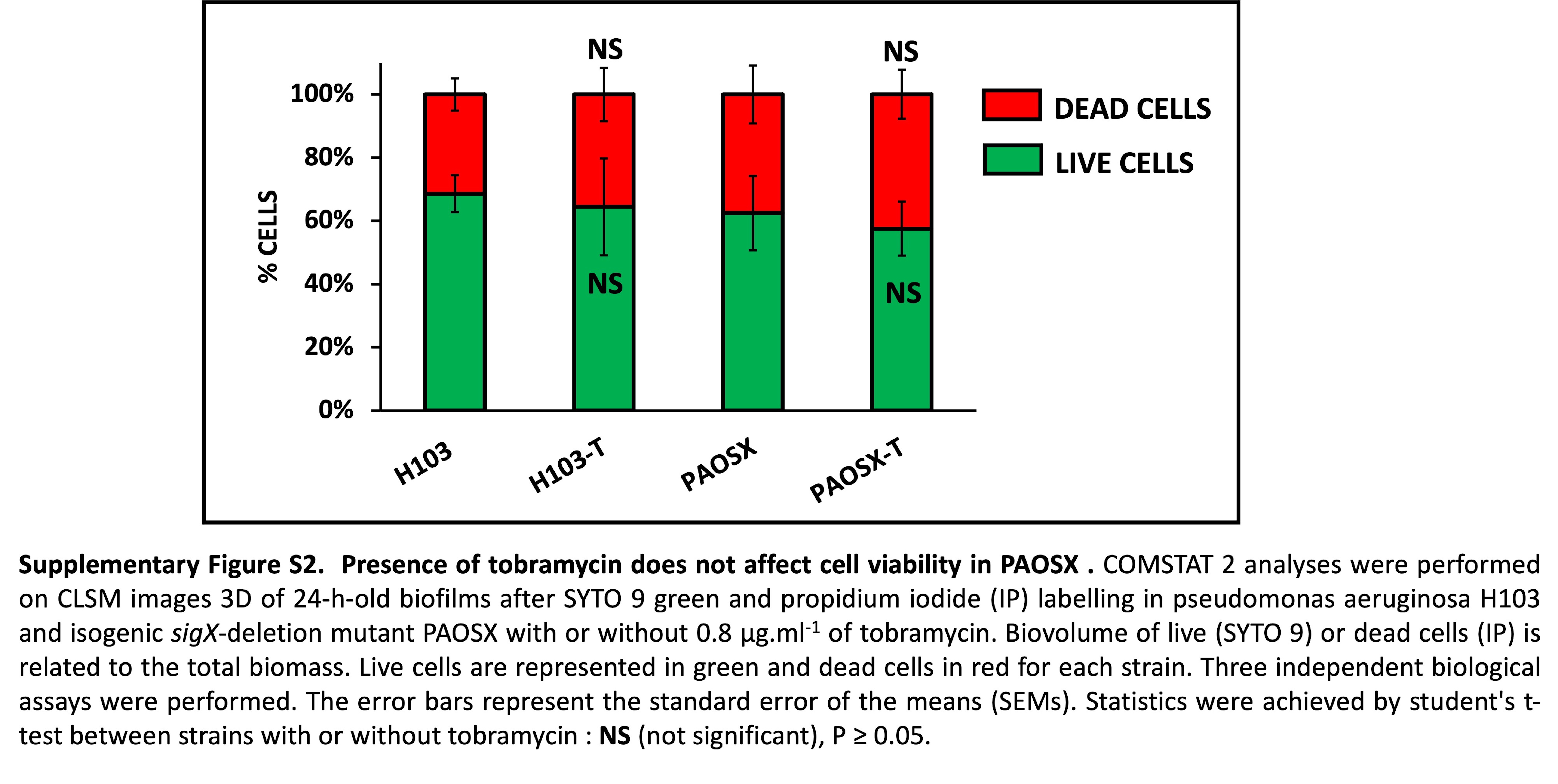

Supplement: Fig. S2 — Presence of tobramycin does not affect cell viability in PAOSX. [file spectrum.02303-23-s0002.tiff]

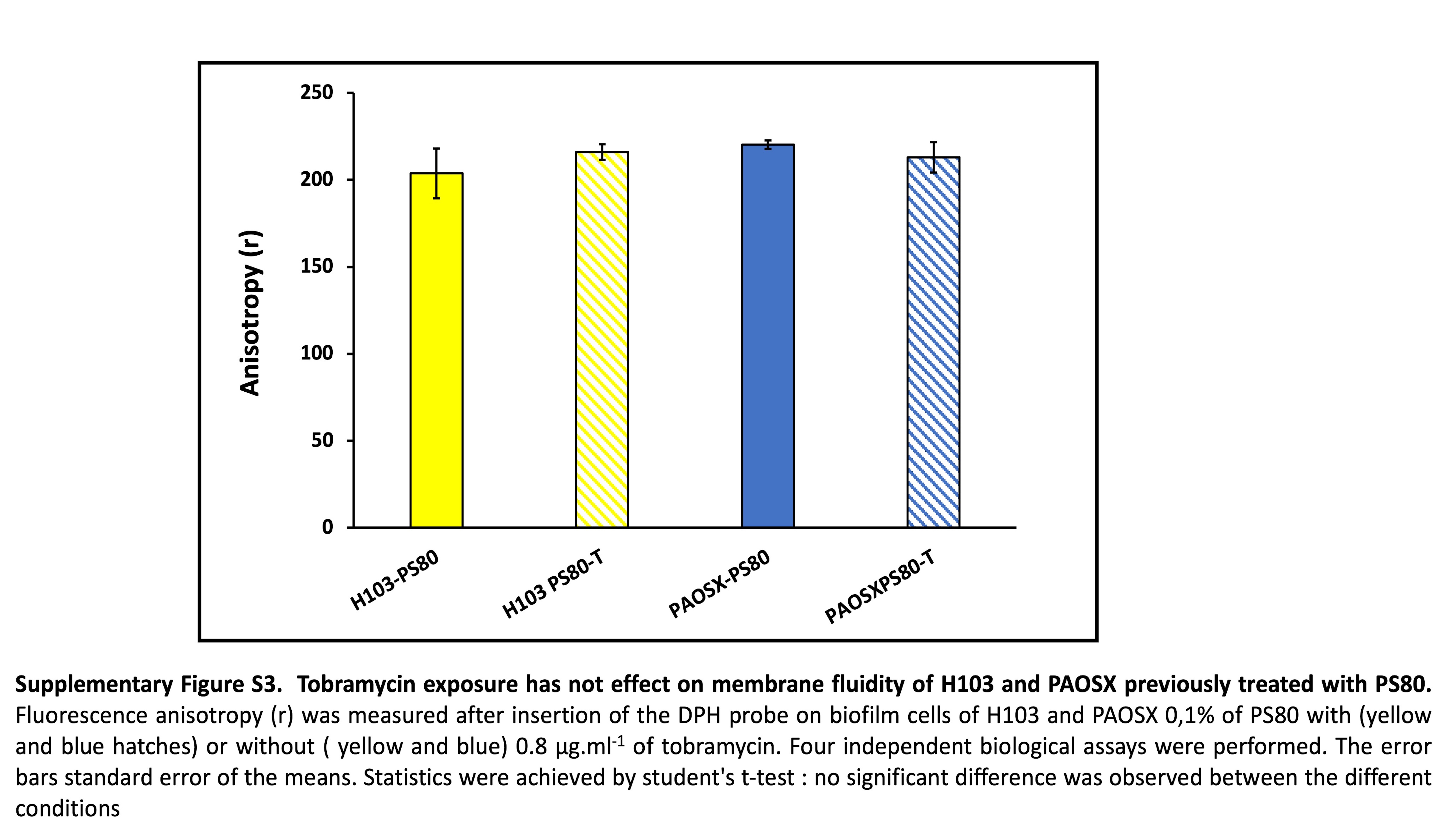

Supplement: Fig. S3 — Tobramycin exposure has no effect on membrane fluidity of H103 and PAOSX previously treated with PS80. [file spectrum.02303-23-s0003.tiff]
