# Supplementary material for: Membrane fluidity homeostasis is required for tobramycin-enhanced biofilm in Pseudomonas aeruginosa
Source: Microbiol Spectr. 2024 Feb 27;12(4):e02303-23. doi: 10.1128/spectrum.02303-23 (PMC10986583; doi:10.1128/spectrum.02303-23)
Supplement: Table S1 — List of strains and primers used in this study. [file spectrum.02303-23-s0004.docx]

**Supplementary Table 1**. List of strains and primers used in this study

Strain Characteristics Reference

H103 Wild-type strain H103; PAO1 prototroph derivative Hancock et Carey, 1979 PAOSX H103 Δ*sigX* Bouffartigues *et al*., 2012

H103(GFP) H103 with pBB686GFP vector This study

qPCR

PA number Gene name Primer name Sequence (5’ - 3’)

PA0762 *algU* F-algU: TACCTGGCTGTATCGGATCG

R-algU: GAAGAACTCCGCATCCTCTG

PA3540 *algD* F-algD: GGGCTATGTCGGTGCAGTATG

R-algD: GCGACTTGCCCTGGTTGAT

PA1776 *sigX* F-sigX: AATTGATGCGGCGTTACCA

R-sigX: CCAGGTAGCGGGCACAGA

PA1774 *cfrX* F-sigX: CTGCGGGACCTCGTCAAG

R-sigX: GCCGACCTGGCGATTG

PA1775 *cmpX* F-cmpX: TATCTGGACCCAGAGCTTGC

R-cmpX: GAAGCCGAGCAGAACGA
